# Is aspirin still relevant as a single pharmacological agent for venous thromboembolism prophylaxis post hip and knee arthroplasty surgery: A retrospective review

**DOI:** 10.1051/sicotj/2022029

**Published:** 2022-06-28

**Authors:** Jacques Pretorius, Nouman Nemat, Imran Azeem, Tony Shaju, Sayed Nadeem, Yasir Hammad

**Affiliations:** Letterkenny University Hospital Kilmacrennan Road, Ballyboe Letterkenny County Donegal F92AE81 Ireland

**Keywords:** Aspirin, Venous thromboembolism prophylaxis, Total hip arthroplasty, Total knee arthroplasty

## Abstract

*Introduction*: Aspirin is widely used for the prevention of venous thromboembolism (VTE) after total hip arthroplasty (THA) and total knee arthroplasty (TKA). It is well-established that the bleeding and wound complication risk for aspirin is low or equivalent to the other anticoagulant agents, but there is still ongoing controversy regarding the efficacy of aspirin for VTE prophylaxis. The current HSE (2012) guideline suggests 72 h of enoxaparin and 4 weeks of Aspirin therapy. But is this practice still relevant with more recent guidelines, NICE (2021) and SIGN (2014) suggesting that Aspirin is not recommended as a single pharmacological agent for VTE prophylaxis. *Method*: A Retrospective review was performed of a single centre, between January 2016 and May 2021 assessing for symptomatic VTE post-THA and TKA. All the patients received enoxaparin and aspirin as per the HSE guidelines. Using NIMIS we assessed which patients received a workup for potential symptomatic VTE and who had a confirmed VTE within 3 months post-surgery. The secondary outcome was mortality within 3 months post-surgery. *Results*: A total of 1178 patients (721 undergoing THR and 457 undergoing TKR) were included in the study. The number of patients who received a workup for potential symptomatic VTE was 124 of 1178 (10.53%). VTE occurred in 13 of 721 patients (1.80%) of the THR patients and 1 of 457 (0.22%) of the TKR patients, and a total of 14 of 1178 patients (1.19%). Of these 7 (0.59%) patients developed a DVT and 7 (0.59%) a PE. No patients suffered a fatal pulmonary embolism within 90 days of index surgery nor any other cause of mortality. *Conclusion*: Aspirin is not only still relevant but can be considered as one of the most optimal pharmacological agents in preventing VTE after THA and TKA.

## Introduction

The venous thromboembolism rate post total hip arthroplasty (THA) and total knee arthroplasty (TKA) is higher than most other orthopaedic surgery, as well as other surgical disciplines [1]. It has been noted that in the absence of thromboprophylaxis, the Deep Venous Thrombosis (DVT) rate for THA is as high as 40–60% and in TKA is 40–85% [2]. This can be reduced to 1–10% with routine thromboprophylaxis agents [2, 3]. Furthermore, the pulmonary embolism rates after THA are 0.9–28% and after TKA is 1.5–25%, with a mortality rate of up to 15% [4–6]. The current health service executive (HSE) guideline recommends mechanical prophylaxis with 72 h of 40 mg enoxaparin, initiated 6–12 h after surgery, and a further 4 weeks of aspirin therapy for patients who received a THA or TKA [7]. This practice is based on recent publications from the American College of Chest Physicians (ACCP) 2012 and the American Academy of Orthopaedic Surgeons (AAOS) 2011 [8, 9]. In our department, we initiate patients on enoxaparin 6–12 h after THA and TKA; we then continue enoxaparin for their hospital stay and discharge patients for a further 4 weeks of 150 mg once daily aspirin therapy. Aspirin, known as acetylsalicylic acid, is a non-steroidal anti-inflammatory drug with anticoagulation effects due to its irreversible inhibition of platelet cyclo-oxygenase and selective inhibition of thromboxane A2 inhibition [10].

But is this practice still relevant with more recent guidelines suggesting that aspirin is not recommended as a single pharmacological agent [11, 12]. The National Institute of Clinical Excellence (NICE) 2021 guidelines for THA recommends either low molecular weight heparin (LMWH) for 10 days followed by 4 weeks of aspirin, or 4 weeks of LMWH, or one of the newer oral anticoagulants (NOAC) [11]. Whereas the Scottish Intercollegiate Guidelines Network (SIGN) 2014 clearly states that aspirin is not recommended as a sole pharmacological agent but recommends LMWH, fondaparinux, rivaroxaban or dabigatran [12]. The question that therefore arises is whether aspirin can be considered an optimal agent for venous thromboembolism (VTE) prophylaxis, in light of these more recent guidelines.

## Materials and methods

This study selected all the patients who received an elective total hip arthroplasty or total knee arthroplasty, in a single center, between January 2016 and May 2021 retrospectively. Letterkenny University Hospital serves the population of Donegal with a catchment area of about 159,000 people [13]. All patients who had already been on Warfarin, Rivaroxaban, Dabigatran, or Fondaparinux prior to surgery were excluded from the study, which included 56 patients as they would continue with their regular anticoagulation therapy instead of receiving aspirin prophylaxis therapy. A total of 1178 patients who received aspirin for VTE prophylaxis were included, of which 721 underwent THA, and 457 received a TKA. This study assessed the rate of VTE as well as mortality for these patients over the first 3 months post-surgery, which was then compared to other similar studies to determine whether our VTE rate is comparable to other studies where a variety of different pharmacological prophylactic agents was used. If this is the case, it will support our statement that aspirin is still relevant as a sole pharmacological agent even with more recent guidelines contradicting this [11, 12].

One thousand one hundred and seventy-eighth patients who underwent a THA and TKA received 40 mg of enoxaparin within 12 h post-surgery and continued with a once-daily dose until they were discharged on aspirin for another 4 weeks [7]. The mean age for THA and TKA arthroplasty was 69.35 (*SD* ± 9.77) and 70.47 (*SD* ± 8.79), respectively. The female to male ratio was 1:1.1. The average length of hospital stay was 5.19 (*SD* ± 7.48) days for THA and 5.09 (*SD* ± 5.68) days for TKA. They also received mechanical prophylaxis with graduated compression stockings up to 6 weeks post the index procedure. The compression stockings were not used if any specific contra-indications were present (peripheral vascular disease, severe congestive cardiac failure, local skin problems). Patients receiving surgery in the morning were mobilized the same day by physiotherapy, and those receiving surgery in the afternoon were mobilized the following day.

The patients with signs and symptoms suggestive of symptomatic VTE would be identified either as an in-patient prior to discharge or presenting to the accident and emergency department after discharge from the hospital. If there is any suspicion of deep venous thrombosis (DVT), patients will undergo a Doppler Compression B-mode Ultrasound scan [14]. All patients with suspected PE received a Computerized Tomographic Pulmonary Arteriogram (CTPA) as this is the imaging of choice with high sensitivity and specificity demonstrated by the PIOPED II trial [15].

In this study the NIMIS system was used, which is a picture archive and communication system (PACS), to achieve our primary outcome in identifying how many patients received a workup (Doppler or CTPA) for potential symptomatic VTE within 90 days and who had a confirmed diagnosis on imaging. This is used as the cut-off by most similar studies as observational data suggests that the incidence of VTE after TKA and THA returns to the presurgical risk level at about 3 months post-surgery [16]. The NIMIS system is widely incorporated throughout Ireland hospital systems and is active in 70 hospitals across Ireland [17]. This meant that we could collect symptomatic VTE data from across the country for the patients who received their index arthroplasty surgery at Letterkenny University Hospital. Our secondary outcome assessed if any patients suffered from a fatal pulmonary embolism or any other cause of mortality within 3 months post-surgery. To establish this, we used the ICM system, which is used by our hospital, through which we could identify if and when a patient has passed away.

## Results

Of the 1178 patients included in the study, a total of 124 (10.53%) patients received a workup for potential symptomatic VTE (Table 1). This would include patients with signs and symptoms suggestive of either a deep venous thrombosis or pulmonary embolism. Patients mainly complained of leg pain, swelling and discomfort or shortness of breath, and chest pain, respectively. Doppler ultrasound was performed on 94 patients with DVT symptoms, and 30 patients received a CTPA. Only 7 of 1178 (0.59%) patients had a confirmed DVT, and 7 patients (0.59%) had a confirmed Pulmonary Embolism (Table 1). There was an overall VTE rate of 14 of 1178 (1.19%) for all arthroplasties performed (Table 1).

**Table 1:** A summary of total number of surgeries performed and how many patients received a workup with a confirmed diagnosis of PE or DVT.

	Total no of operations	No of CTPA performed	No of Doppler U/S	Confirmed PE	Confirmed DVT	Total no of VTE
THA	721	25 (3.47)	55 (7.63)	7 (0.97)	6 (0.83)	13 (1.88)
TKA	457	5 (1.09)	39 (8.53)	0	1 (0.22)	1 (0.22)
Total	1178	30 (2.55)	94 (7.98)	7 (0.59)	7 (0.59)	14 (1.19)

**Table 2:** Summary of key studies included in the study.

Study	Study type	Intervention	Sample size	Findings
Anderson et al. [27]	Randomized Controlled Trial	Multicentre, double-blind RCT involving patients who were undergoing THA/TKA. All the patients received once-daily oral rivaroxaban (10 mg) until postoperative day 5 and then were randomly assigned to continue rivaroxaban or switch to aspirin (81 mg daily).	3424 THA and TKA	Extended prophylaxis with aspirin was not significantly different from rivaroxaban in the prevention of symptomatic venous thromboembolism
Anderson et al. [28]	Randomized Controlled Trial	Multicentre randomized, controlled trial with a non-inferiority design. After an initial 10 days of dalteparin prophylaxis after elective THA, patients were randomly assigned to 28 days of dalteparin or aspirin.	778 THA	Extended prophylaxis for 28 days with aspirin was non-inferior to and as safe as dalteparin for the prevention of VTE
Warren et al. [22]	Registry study (National Surgical Quality Improvement Program database)	The American College of Surgeons NSQIP database identified 363,530 patients who received a TKA or THA from 2008 to 2016. Bimodal multivariate logistic regression models for THA and TKA were developed for 2009–2016 using 2008 as a reference.	363,530 THA and TKA	Overall VTE rate for THA and TKA was 0.6% and 1.4%, respectively within 30 days post-surgery
Pedersen et al. [26]	15-year retrospective cohort study	The risk of thrombotic and major bleeding events in patients undergoing total hip and knee replacement (THR and TKR) treated with thromboprophylaxis, using nationwide population-based databases.	83,756 THA and TKA	A VTE rate of 1.3% for THA and 1.5% for TKA
Fuji et al. [25]	Retrospective analysis of a Japanese healthcare database	The study comprised 36,947 patients who had undergone orthopedic surgeries of the lower extremities, with the source population of the database being derived from 100 acute-care hospitals with diagnosis procedure combination.	36,947 THA and TKA	An overall VTE rate of 1.4%
Wells et al. [33]	A retrospective study	A retrospective study was conducted using a US health plan claims database linked to an in-patient database containing medication use. Outcomes were compared using χ^2^ tests; predictors of outcomes were analyzed using multivariate logistic regression.	3497 THA and TKA	Higher VTE rate in patients receiving anticoagulation therapy for less than 14 days (3.9%) compared to more than 14 days (1.4%)
Faour et al. [35]	A retrospective study	Exploratory univariate analyses were used to compare confounders between the study groups. Multivariate regression was used to control for confounding variables.	7488 THA	No difference in the incidence of symptomatic VTE after THA with low-dose (81 mg) compared with standard-dose aspirin (325 mg)

The overall VTE, PE, and DVT rate for THA was 1.8%, 0.97%, and 0.83% (13, 7, and 6 out of 721), respectively, whereas the overall VTE, PE, and DVT rate for TKA patients was 0.22%, 0%, and 0.22% (1, 0, and 1 out of 457), respectively (Table 1).

The mean time to presentation was 44.43 days (*SD* ± 32.59) for the symptomatic DVT patients and 24.57 days (*SD* ± 13.43) for the symptomatic PE patients.

No patients suffered a fatal pulmonary embolism within 90 days of index surgery nor any other cause of mortality.

## Discussion

It is well-established that some of the major causes of readmission post THA and TKA are patients developing symptomatic DVT and PE [18]. For this reason, it is imperative that we, as orthopedic surgeons have an in-depth knowledge of pathophysiology and potential treatment modalities to prevent this. Many retrospective and prospective studies have been performed to identify the ideal pharmacological agent for VTE prophylaxis but there continues to be controversy regarding the optimal agent after joint arthroplasty [19]. A number of national guidelines are available and attempt to give guidance to orthopedic surgeons as to which agent is most effective [7, 11, 12]. The HSE guidelines advise prolonged low-dose aspirin therapy, which is not advised as a single pharmacological agent by the NICE and SIGN guidelines, but rather LMWH, Fondaparinux, Rivaroxaban, Apixaban, or Dabigatran [11, 12]. SIGN argues that there is sufficient data available to support the fact that aspirin is inferior to other anticoagulants [12]. A pharmacological agent can be considered an optimal treatment modality if it meets the following criteria: high efficacy in VTE prevention, low risk of bleeding, easily administered, cost-effective, and decreased postoperative complications [20].

Aspirin is easily administered, readily available and the most inexpensive of the comparative agents. Aspirin is an effective agent in preventing ischaemic cardiovascular and cerebrovascular disease, however, there remains controversy about whether aspirin is an effective drug for VTE prophylaxis post arthroplasty surgery [21]. We aimed to add data to support that aspirin is in fact a suitable agent.

Our local overall VTE rate for a 90-day follow-up period for 1178 arthroplasties performed was 1.19%. This is comparable to other large retrospective databases [22–25]. One of the largest databases was the American College of Surgeons National Surgical Quality Improvement Program (NSQIP) database which identified 363,530 patients who received a TKA or THA from 2008 to 2016. The overall VTE rate for THA and TKA was 0.6% and 1.4% respectively within 30 days post-surgery [22]. The numbers will likely be higher if a 90-day follow-up period was used as our mean time to presentation for symptomatic DVT was 44 days. A large Danish Hip Arthroplasty registry between 1997 and 2011 found a VTE rate of 1.3% for THA and 1.5% for TKA [26]. In Japan an administrative database of patients from 2008 to 2013 had an overall VTE rate of 1.4% [25]. The UK National Joint Registry (NJR) reports of 2010 and 2007 showed 90-day PE rates of 0.68% and 0.6%, respectively, post-THA and 0.6% post-TKA in the 2007 report [23, 24]. In our study, the overall PE rates post THA and TKA was 0.59% which is comparable to the UK NJR.

There have also been multiple direct comparative studies of aspirin versus Rivaroxaban and LMWH, suggesting that aspirin is as effective in preventing VTE post arthroplasty surgery [27, 28]. The EPCAT I trial (Extended Prophylaxis Comparing Low-Molecular-Weight Heparin to Aspirin in Total Hip Arthroplasty) suggested that the extended course of aspirin is non-inferior to extended LMWH [28]. The EPCAT II trial, which enrolled 3424 patients, also confirmed no statistically significant difference in outcome comparing Aspirin versus Rivaroxaban [27].

It has already been well established that the bleeding and wound complication risk of aspirin is comparable to or even better than most other anticoagulants used for VTE prophylaxis. Zou et al. stated that rivaroxaban had increased postoperative hidden blood loss, and postoperative wound complications [29]. This was also the case in the EPCAT I trial where aspirin was found to have a better bleeding risk rate than LMWH [28]. Multiple studies have cited a low risk of bleeding among patients receiving aspirin post total joint arthroplasty [20, 30–32].

The HSE guidelines suggest 150 mg of aspirin once daily for 4 weeks [7]. The importance of prolonged anticoagulation therapy was underlined in a retrospective study performed by Wells et al. that indicated a much higher VTE rate in patients receiving anticoagulation therapy for less than 14 days (3.9%) compared to more than 14 days (1.4%) [33]. Similarly, a systematic review comparing low dose (<162 mg/day) to high-dose (>162 mg/day) aspirin in patients post total joint arthroplasty found no statistically significant difference in terms of DVT, PE, 90-day mortality, and major bleeding [34]. Two large retrospective reviews performed recently supported this finding that low dose of aspirin (81 mg) had a similar outcome to a high dose of aspirin (325 mg) in THA and TKA patients [35, 36].

Graduated compression stockings are also provided to the patients as per the HSE guidelines, and their benefits have been undeniably supported by a study performed by Agu et al. which showed a 57% reduction of DVT post-THA [37].

The results of this study support the fact that low dose, prolonged duration aspirin with appropriate mechanical prophylaxis is comparable to the other anticoagulation therapy advised by the NICE and SIGN guidelines [11, 12].

But one of the limitations to this study is that our hospital technically uses combined pharmacological treatment, with enoxaparin given as an in-patient and then changed to aspirin therapy on discharge. The use of combined pharmacological therapy was supported by the EPCAT II trial, which showed that no statistically significant difference in VTE risk after TKA and THA if aspirin was given after an initial 5 days of rivaroxaban [27]. A recent review of the use of aspirin after joint arthroplasty concluded that for appropriately selected low-risk patients, aspirin is effective following initial rivaroxaban therapy [38]. The EPCAT III trial is an ongoing trial that will focus on comparing aspirin as a solo pharmacological agent to a combined regimen which will give us better clarity if the VTE rate would still be comparable without the initial transition phase. As stated by Fontalis et al., it is essential to accurately risk stratify patients into low-intermediate-high individuals as this will allow us to adjust our prophylaxis strategy accordingly [39] and therefore improve our overall VTE rates.

## Conclusion

Aspirin is not only still relevant but can be considered one of the most optimal pharmacological agents in preventing VTE after THA and TKA. Aspirin has an efficacy comparable to the other anticoagulation drugs, has a low bleeding and wound complication risk, is easily administered, and is cost-effective.

## Importance of article

There is a major risk of developing VTE post total knee and hip arthroplasty. There is still ongoing controversy regarding the optimal prophylactic agent to be used. Aspirin is an inexpensive, readily available agent used for a long time by orthopedic surgeons for VTE prophylaxis. But confidence in aspirin has been waning recently, with national guidelines like NICE and SIGN suggesting that it should not be used as a single pharmacological agent. The Irish HSE guideline still suggests the use of aspirin therapy – as is the case in our hospital. Our review of 1178 patients who underwent a THA and TKA suggests that aspirin is still relevant for use in VTE prophylaxis and can even be considered one of the most optimal agents.

## References

[1] White R, Zhou H, Romano P (2003) Incidence of symptomatic venous thromboembolism after different elective or urgent surgical procedures. Thromb Haemost 90, 446–455. 12958614 10.1160/TH03-03-0152

[2] Baser O (2011) Prevalence and economic burden of venous thromboembolism after total hip arthroplasty or total knee arthroplasty. Am J Manag Care 17, S6–8. 21517654

[3] Knesek D, Peterson TC, Markel DC (2012) Thromboembolic prophylaxis in total joint arthroplasty. Thrombosis 2012, 1–8. 10.1155/2012/837896 PMC3458274 23029611

[4] Geerts WH, Bergqvist D, Pineo GF, et al. (2008) Prevention of venous thromboembolism. Chest 133, 381S–453S. 18574271 10.1378/chest.08-0656

[5] Januel J-M, Chen G, Ruffieux C, et al. (2012) Symptomatic in-hospital deep vein thrombosis and pulmonary embolism following hip and knee arthroplasty among patients receiving recommended prophylaxis. JAMA 307 (3), 294–303. 22253396 10.1001/jama.2011.2029

[6] Goldhaber SZ, Visani L, de Rosa M (1999) Acute pulmonary embolism: Clinical outcomes in the International Cooperative Pulmonary Embolism Registry (ICOPER). Lancet 353, 1386–1389. 10227218 10.1016/s0140-6736(98)07534-5

[7] Health Service Executive (HSE) (2012) Suggested guideline for thromboprophylaxis post total hip arthoplasty (THA) and total knee arthroplasty (TKA). https://www.hse.ie/eng/services/publications/clinical-strategy-and-programmes/guideline-for-thromboprophylaxis-post-total-hip-arthoplasty-and-total-knee-arthroplasty.pdf. Accessed 7 Mar 2022.

[8] Falck-Ytter Y, Francis CW, Johanson NA, et al. (2012) Prevention of VTE in orthopedic surgery patients. Chest 141, e278S–e325S. 22315265 10.1378/chest.11-2404 PMC3278063

[9] The American Academy of Orthopaedic Surgeons (2011) Preventing venous thromboembolic disease in patients undergoing elective hip and knee arthroplasty. https://www.aaos.org/quality/quality-programs/tumor-infection-and-military-medicine-programs/venous-thromboembolic-disease-in-elective-tka-and-tha-prevention/. Accessed 19 Feb 2022. 10.5435/00124635-201112000-00007 22134209

[10] Catella-Lawson F (2001) Vascular biology of thrombosis: Platelet-vessel wall interactions and aspirin effects. Neurology 57, S5–S7. 10.1212/wnl.57.suppl_2.s5 11552047

[11] National Institute for Health and Care Excellence (2021) Reducing venous thromboembolism risk: Orthopaedic surgery [NICE pathway]. https://pathways.nice.org.uk/pathways/venous-thromboembolism/reducing-venous-thromboembolism-risk-orthopaedic-surgery#path=view%3A/pathways/venous-thromboembolism/reducing-venous-thromboembolism-risk-orthopaedic-surgery.xml&content=view-index. Accessed 03 Jan 2022.

[12] Scottish Intercollegiate Guidelines Network (SIGN) (2014) Prophylaxis of venous thromboembolism. Edinburgh, SIGN. (SIGN publication no. 122). https://www.sign.ac.uk/our-guidelines/prevention-and-management-of-venous-thromboembolism/. Accessed 10 Jan 2022.

[13] The Friends of Letterkenny University Hospital (2022) About Letterkenny University Hospital. https://thefriends.ie/letterkenny-university-hospital/. Accessed 19 Feb 2022.

[14] Gudmundsen TE, Vinje B, Pedersen T (1990) Deep vein thrombosis of lower extremities. Acta Radiologica 31, 473–475. 2261293

[15] Gottschalk A, Stein PD, Goodman LR, Sostman HD (2002) Overview of prospective investigation of pulmonary embolism diagnosis II. Semin Nucl Med 32, 173–182. 12105798 10.1053/snuc.2002.124177

[16] Bjørnarå BT, Gudmundsen TE, Dahl OE (2006) Frequency and timing of clinical venous thromboembolism after major joint surgery. J Bone Joint Surg Br 88 (B), 386–391. 16498018 10.1302/0301-620X.88B3.17207

[17] Implementing the National Integrated Medical Imaging System (NIMIS). https://www.changehealthcare.ie/insights/implementing-the-national-integrated-medical-imaging-system. Accessed 22 Feb 2022.

[18] Freedman KB, Brookenthal KR, Fitzgerald RH, et al. (2000) A meta-analysis of thromboembolic prophylaxis following elective total hip arthroplasty. J Bone Joint Surg Am 82, 929–938. 10901307 10.2106/00004623-200007000-00004

[19] Raphael IJ, Tischler EH, Huang R, et al. (2014) Aspirin: An alternative for pulmonary embolism prophylaxis after arthroplasty? Clin Orthop Rel Res 472, 482–488. 10.1007/s11999-013-3135-z PMC3890197 23817755

[20] Agaba P, Kildow BJ, Dhotar H, et al. (2017) Comparison of postoperative complications after total hip arthroplasty among patients receiving aspirin, enoxaparin, warfarin, and factor Xa inhibitors. J Orthop 14, 537–543. 28878512 10.1016/j.jor.2017.08.002 PMC5574820

[21] Brown GA (2009) Venous thromboembolism prophylaxis after major orthopaedic surgery: A pooled analysis of randomized controlled trials. J Arthroplasty 24, 77–83. 10.1016/j.arth.2009.06.002 19628366

[22] Warren JA, Sundaram K, Anis HK, et al. (2020) Have venous thromboembolism rates decreased in total hip and knee arthroplasty? J Arthroplasty 35, 259–264. 31530463 10.1016/j.arth.2019.08.049

[23] Emsley D, Martin J, Newell C, et al. (2008) National Joint Registry for England and Wales: 5th Annual Report National Joint Registry Part 1. The NJR Steering Committee.

[24] Ellams D, Forsyth O, Mistry A, et al. (2010). 7th Annual Report: National Joint Registry for England and Wales. URL https://reports.njrcentre.org.uk/Downloads. Accessed 7 March 2022.

[25] Fuji T, Akagi M, Abe Y, et al. (2017) Incidence of venous thromboembolism and bleeding events in patients with lower extremity orthopedic surgery: A retrospective analysis of a Japanese healthcare database. J Orthop Surg Res 12, 55. 28376907 10.1186/s13018-017-0549-4 PMC5381070

[26] Pedersen AB, Mehnert F, Sorensen HT, et al. (2014) The risk of venous thromboembolism, myocardial infarction, stroke, major bleeding and death in patients undergoing total hip and knee replacement. Bone Joint J 96 (B), 479–485. . 24692614 10.1302/0301-620X.96B4.33209

[27] Anderson DR, Dunbar M, Murnaghan J, et al. (2018) Aspirin or rivaroxaban for VTE prophylaxis after hip or knee arthroplasty. N Engl J Med 378, 699–707. 29466159 10.1056/NEJMoa1712746

[28] Anderson DR, Dunbar MJ, Bohm ER, et al. (2013) Aspirin versus low-molecular-weight heparin for extended venous thromboembolism prophylaxis after total hip arthroplasty. Ann Intern Med 158, 800. 23732713 10.7326/0003-4819-158-11-201306040-00004

[29] Zou Y, Tian S, Wang Y, Sun K (2014) Administering aspirin, rivaroxaban and low-molecular-weight heparin to prevent deep venous thrombosis after total knee arthroplasty. Blood Coagul Fibrinolys 25, 660–664. 10.1097/MBC.0000000000000121 24695091

[30] Lotke PA, Lonner JH (2006) The benefit of aspirin chemoprophylaxis for thromboembolism after total knee arthroplasty. Clin Orthop Rel Res 452, 175–180. 10.1097/01.blo.0000238822.78895.95 16957642

[31] Reitman RD, Emerson RH, Higgins LL, Tarbox TR (2003) A multimodality regimen for deep venous thrombosis prophylaxis in total knee arthroplasty. J Arthroplasty 18, 161–168. 12629605 10.1054/arth.2003.50026

[32] Santana DC, Emara AK, Orr MN, et al. (2020) An update on venous thromboembolism rates and prophylaxis in hip and knee arthroplasty in 2020. Medicina (Lithuania) 56, 1–14. 10.3390/medicina56090416 PMC7558636 32824931

[33] Wells PS, Borah BJ, Sengupta N, et al. (2010) Analysis of venous thromboprophylaxis duration and outcomes in orthopedic patients. Am J Manag Care 16, 857–863. 21348557

[34] Azboy I, Groff H, Goswami K, et al. (2020) Low-dose aspirin is adequate for venous thromboembolism prevention following total joint arthroplasty: A systematic review. J Arthroplasty 35, 886–892. 31733981 10.1016/j.arth.2019.09.043

[35] Faour M, Piuzzi NS, Brigati DP, et al. (2019) No difference between low- and regular-dose aspirin for venous thromboembolism prophylaxis after THA. Clin Orthop Rel Res 477, 396–402. 10.1097/CORR.0000000000000613 PMC6370078 30624322

[36] Faour M, Piuzzi NS, Brigati DP, et al. (2018) Low-dose aspirin is safe and effective for venous thromboembolism prophylaxis following total knee arthroplasty. J Arthroplasty 33, S131–S135. 29656974 10.1016/j.arth.2018.03.001

[37] Agu O, Hamilton G, Baker D (2002) Graduated compression stockings in the prevention of venous thromboembolism. Br J Surg 86, 992–1004. 10.1046/j.1365-2168.1999.01195.x 10460633

[38] Kahn SR, Shivakumar S (2020) What’s new in VTE risk and prevention in orthopedic surgery. Res Pract Thromb Haemost 4, 366–376. 32211571 10.1002/rth2.12323 PMC7086463

[39] Fontalis A, Berry DJ, Shimmin A, et al. (2021) Prevention of early complications following total hip replacement. SICOT J 7, 61. 34851264 10.1051/sicotj/2021060 PMC8634898

